# Translating Ethical Principles into Law, Regulations and Workable Animal Welfare Practices

**DOI:** 10.3390/ani15060821

**Published:** 2025-03-13

**Authors:** David J. Mellor, D. Mette Uldahl

**Affiliations:** 1 Animal Welfare Science and Bioethics Centre, School of Veterinary Science, Massey University, Palmerston North 4442, New Zealand; 2 Vejle Equine Practice, Fasanvej 12, 7120 Vejle Oest, Denmark; mette@uldahl.eu

**Keywords:** ethics, Utilitarianism, Animals Rights, anti-cruelty, animal-protection and animal-welfare or animal-care laws, change in welfare concepts, sentience, suffering, positive states, Five Domains Model, science-ethics integration, national, international

## Abstract

The ethical theories considered in this commentary include Anthropocentrism, Dominionism, Utilitarianism, Reverence for Life, Animal Rights, Biocentrism, Ecocentrism and Care Ethics. It is apparent that Utilitarianism provides a motivating rationale for devising legal instruments to manage animal welfare in many countries. The emphasis of different laws spanning many decades paralleled the trajectory of changing attitudes to animals. Initial laws focussed on serious abuse, and were enacted to deal with relative indifference to animal suffering. Anticruelty laws followed; they dealt with a wider range of noxious acts that also cause suffering. Animal Protection laws accommodated a growing acceptance that much less severe, yet still very unpleasant experiences, are of significant welfare concern. These laws and their amendments, plus the associated instruments (e.g., Codes of Welfare/Practice), were increasingly couched using animal welfare terminology. Finally, contemporary laws that focus directly to animal welfare, increasingly refer to a ‘duty of care’ towards animals, entailing responsibilities to minimise negative experiences and to promote positive ones. Outlined here is an example of an ethically-based legal structure for providing guidance and outlining requirements for animal welfare management nationally. It has four interacting levels: Level 1—Law; Level 2—Codes of Welfare/Practice; Level 3—Regulations; and Level 4—Cooperation at a National Level. It is noted that although the framing of this legal structure is based on ethical principles, the expression of those principles is more implicit than explicit. However, expression of the ethical principles can be made much more explicit when putting into effect legal requirements to assess the acceptability of different practices in animal welfare terms. There are four interacting steps in this process: Step 1—the Primary Assumption: Animals have intrinsic value and an interest in having good lives. Step 2—Distribution of Responsibility: Humans usually control human-animal interactions and have an overarching responsibility to operate two principles: (1) If in doubt err on the side of the animal; (2) If there are justified concerns, a lack of evidence can never in itself justify a practice. Step 3—Assessment of Impact on the Animals: Use a structured framework to assess the impact of human-initiated activities. The Five Domains Model is suggested as a suitable device for this. Step 4—Rigorous Evaluation: the aim, to choose the least noxious intervention as required by the Principle of Proportionality which, once chosen, supports a justification to proceed. This commentary provides examples of how ethical reasoning, and its operational consequences, can be made visible at every stage of developing, introducing and operating infrastructures for managing animal welfare nationally.

## Simple Summary

This commentary begins by noting that the ethics of human domination over animals for our own purposes reveals the foundational values that sustain our drive to minimise the welfare harms that very often attend that domination. At one extreme, claims of an absolute right to do anything that benefits people no matter what the cost to the animals, are greatly undermined by their apparent callousness. On the other hand, Utilitarian ethics which has been widely adopted internationally, asserts that for an action to be acceptable ethically, all of the harms must be outweighed by all of the benefits. Noted here, however, is that the “Strong Utilitarian” version should be used: namely, that conscientious and comprehensive attention must be given both to identifying and minimising all possible harms and to identifying and maximizing all realistically anticipated benefits; also, that action must be taken to ensure that the separation between the two is the greatest that can be realistically achieved. At the other extreme is Animal Rights ethics, which claims that any use of animals for human purposes is exploitative and not acceptable. It is noteworthy, however, that there are some significant benefits when Animal Rights ideas are made available for the public to consider. Care Ethics, which focuses on emotional responses to animals in need and resists actions that cause them harm, harmonises with Animal Rights objectives, but has different starting assumptions. This commentary continues with an account of how, during the last 40 years, scientific understanding of animal welfare has developed as the original influential concepts were modified, extended or replaced. This updated and enhanced the body of welfare knowledge to the extent that Animal Welfare Science is now well established as a distinct discipline. Understanding this welfare journey provides insights into parallel developments in legal instruments related to human uses of animals. The rest of the commentary seeks to show how ethical reasoning, integrated with scientific understanding, provides a coherent platform for formulating and operating effective legal structures to promote acceptable standards of animal welfare nationally. Once shown to be efficacious nationally, they are often considered for international adoption.

## 1. Introduction

It is important to clarify which animals are the focus of this commentary. The generic term ‘animals’ here refers to non-human animals, and among them it refers to those that are sentient. This means animals that are functionally capable of consciously having negative and/or positive subjective experiences which matter to them [1]. Present scientific evidence supports the inclusion of vertebrates and some invertebrates as sentient, although the marginal limits to sentience among invertebrates are still being discussed. Yet even among vertebrates, knowledge of some may be insufficient to understand how any unique sensory characteristics they possess (e.g., echolocation in cetaceans) might be integrated into their expression of sentience.

Section 3 refers to early anticruelty laws being focused on named groups of animals subjected to specific forms of cruelty or abuse, and other laws which sought to make often-conducted cruel processes more humane. The tight focus of statutes at that early stage and thereafter for some time, likely reflected a need for those affected to be in no doubt that the law applied to them. Also, in being so clearly defined in content and circumscribed in application, i.e., keeping the message simple at a time of widespread indifference to animal suffering, this might also have aided the passage of these statues through parliament.

Today, after at least four decades of active scientific endeavour to elucidate the dimensions of animal welfare and how welfare can be assessed and managed (outlined in Section 4), the wider public in many countries now view reasonable standards of animal welfare to be, at the very least, “a good thing”, even when they do not fully understand the background details. One outcome of this is that animal welfare legislation has become more generic.

In New Zealand, for example, the Animal Welfare Act 1999 [2] applies to all those in the country who own or who are in charge of an animal, and it requires them to exercise a “duty of care” by providing for the physical, health and behavioural needs of the animal. Furthermore, the Act, which was passed with unanimous support from parliamentarians, applies to a very wide range of animals. As stipulated [3], “*animal* means any live member of the animal kingdom, that is: a mammal; or a bird; or a reptile; or an amphibian; or a fish (bony or cartilaginous); or any octopus, squid, crab, lobster, or crayfish (including freshwater crayfish); or any other member of the animal kingdom which is declared from time to time by the Governor-General, by Order in Council, to be an animal for the purposes of this Act; and includes any mammalian foetus, or any avian or reptilian pre-hatched young, that is in the last half of its period of gestation or development; and includes any marsupial pouch young; but does not include: a human being; or … any animal in the pre-natal, pre-hatched, larval, or other such developmental stage”.

More specific indications of the diverse species covered by the Act may be illustrated by those used for research, teaching and testing (RTT). In New Zealand they include [4]: horses, cattle, deer, goats, sheep, pigs, domestic poultry, game birds, other birds including endemic, native and introduced species, dogs, cats, guineapigs, mice, rats, rabbits, ferrets, stoats, weasels, wallabies, possums, cetaceans, reptiles, amphibians and fishes. The RTT purposes have included fundamental and applied biomedical, veterinary, agricultural, ecological, welfare and other areas of scientific interest. Beyond the laboratory, additional areas include negative and positive welfare impacts of proposed new or modified approaches to housing, managing and/or interacting with farm, working, sport, companion, zoo, entertainment, research, ‘pest’, free-living, wildlife animals, and exotics including whales (for references see [4]).

Implicit in the commentary thus far is the general point that humans dominate the relationship with animals by influencing their lives and living conditions. We do this either directly via purposeful interactions or indirectly via widespread human impacts on their environments. The pervasiveness of human dominance poses challenging questions, some of which are considered in this commentary (Section 2).

How can the levels of imposed animal welfare compromise be justified as acceptable? Do we humans have a fundamentally inherent privilege or right, simply by virtue of being human, to impose harmful acts on animals where we judge the acceptability of the noxious outcomes against our own self-interest? On the other hand, can our conceptual frameworks regarding animals’ intrinsic value or moral equivalence, when combined with the precautionary principle of “when in doubt err in favour of the animals”, provide sufficient ethical justification to support undertaking welfare compromising animal uses that serve our interests? What compromises can never be justified? And finally, at a governance level, what principles are available to underpin laws and regulations designed to guide, define and promote the transparent care, protection and management of animals focussed on their welfare?

These matters are evaluated under the following sections headings. Section 2: Some ethical perspective on animal welfare. Section 3: The emergence of humane societies and laws against animal abuse or cruelty. Section 4: Changes in Animal Welfare Science thinking 1980 to 2024. Section 5: The prelude to scientists supporting the notion that many animals are sentient. Section 6: Legal recognition of animal sentience. Section 7: Implementing ethical principles in national and international law. Section 8: Translating ethical principles into workable animal welfare practices. Section 9: The Five Domains Model for detailed animal welfare assessment. Section 10: The Principle of Proportionality. Section 11: Concluding Comments.

## 2. Some Ethical Perspectives on Animal Welfare

There are several ethical perspectives that have addressed the idea that humans have the prerogative to use animals in ways that compromise their welfare.

*Anthropocentrism*, in general terms sees humankind as separate from nature and superior to it, and other entities including animals, plants and minerals are viewed as resources for human use [5]. Thus, it is argued that animals can be used or compromised if this benefits humanity, as humans are taken to be the most important or morally superior beings. In these terms, if actions serve human survival, progress or convenience they are justified.

*Dominionism* is a form of anthropocentrism, which, when focused specifically on the present context, asserts that human beings have a divinely ordained or otherwise unquestionable right to use animals and everything else in the living world for their own benefit [6]. However, this does not exclude the responsibility to be caring guardians.

*Utilitarianism (Consequentialism)* is an ethical framework that evaluates actions based on their consequences, aiming to maximise overall happiness or utility and to minimise harms [7]. In general terms, it is a response to the question, “How can I make the world a better place?” Compromising the welfare of animals might be justified if the benefits of doing so outweigh the harms caused to the animals. However, Utilitarianism in practice can be weak or strong [8,9]. *Weak Utilitarianism* involves providing only vague “lip-service” justifications expressed as nonspecific benefits whilst giving little attention to identifying and minimising any harms.

On the other hand, *Strong Utilitarianism* demands that conscientious and comprehensive attention be given both to identifying and maximising all realistically possible benefits and to identifying and minimising all possible harms. Note that conscientious minimisation of harms must be applied over the full spectrum from very low anticipated harm to very high; it does not apply only when anticipated harms are high (see Section 10). The aim: to ensure that the separation between all such benefits and all such harms is the maximum that can realistically be achieved. Only then should a judgement be made about whether to continue a current practice or introduce a new one.

It is argued here that for Utilitarianism to be justified ethically, meeting the requirements of its strong variety should be understood to be a moral obligation. Likewise, when an animal welfare statute requires all owners or persons in charge of an animal to exercise a “duty of care” to meet the animal’s physical, health and behavioral needs, for example, as in New Zealand’s Animal Welfare Act 1999 [10], this implies both moral and legal obligations. Introducing moral obligations to Consequentialist reasoning somewhat blurs a distinction between that and Animal Rights reasoning which is mainly focuses on moral principles representing obligations and not consequences (see below).

Interestingly, Jeremy Bentham (1748–1832), often regarded as the founder of Utilitarianism, introduced the idea that there is no justification for treating non-human animals worse than humans [11]. He also stated: “The question is not can they reason, nor can they think, but can they suffer. Why should the law refuse its protection to any sensitive being?”

Singer advanced the idea that the interests of animals and people should be given equal consideration. He popularised the term *speciesism*, coined by Richard Ryder in 1970, to challenge unconstrained anthropocentrism by presenting it as a form of prejudice like racism or sexism [12,13]. Regarding what can be justified within the Utilitarian framework, he opposed all avoidable human-induced suffering.

*Reverence for Life*: Albert Schweitzer (1875–1965) included a strong spiritual dimension in his philosophy of “reverence for life” [14,15]. He viewed benevolence to be a primary aim, a motivation to strive in all our actions in the world to improve the lot of the beings around us, be they humans, animals or plants. Lives may be lost, but they should never be sacrificed in a callous or cavalier manner, and any act of potential harm should be undertaken only after determining that the potential greater good exceeds the harm that occurs from loss of life.

*Animal Rights Theory* counters human superiority. It provides deontological perspectives that focus on moral principles representing obligations and not consequences [1,9,12]. In general terms, Rights Theory is a response to the question, “What sorts of rules apply?” Thus, it asserts that all beings have an inherent right to exist on their own terms independently of their usefulness to others; also, that animals should not be treated as property or resources. This is an absolutist perspective, where merely modifying existing systems is seen as unacceptable. Only abolition of practices that compromise animals would receive approval, as all of them contravene the moral imperatives of the theory. Animal Rights Theory therefore asserts that it is not acceptable to use animals for any human purpose, and that any such use is exploitative.

*Biocentrism* asserts the principle that all forms of life (animals, plants, ecosystems) have moral worth and deserve respect, regardless of the species or its utility to humans. It espouses rights tenets for animals, noting that these principles have primacy when considering the interests and welfare of animals [16].

*Ecocentrism* is an environmental philosophy that goes beyond inclusion of sentient beings and assigns intrinsic value to both individual living beings and entire ecosystems, regardless of their utility to humans. It emphasises that ecosystems, natural processes and species have inherent worth and should be respected and protected for their own sake. It can be seen as an extended form of both Animal Rights Theory and Biocentrism, and is in opposition to Anthropocentrism [17].

*Animal Welfare Theory* is mainly Utilitarian and therefore focuses on consequences. It asserts that using animals for human purposes may be justifiable when the claimed necessity of each purpose survives rigorous questioning, and when the methods are demonstrably the most humane, or rarely for really pressing situations, the least inhumane available for achieving those purposes.

There is a genuine difference in assuming a starting point where humans cannot use animals as resources or for any other purposes, and assuming that human interactions with and use of animals are acceptable. As such this serves as a dividing line between philosophies.

In view of the contrast between Animal Rights and Animal Welfare Theories, it is not likely that Rights Theory will be widely adopted as a basis for human-animal interactions at a national level, or in federal systems, at state or provincial levels. Nevertheless, Animal Rights advocacy has demonstrably succeeded in limiting animal suffering by promoting the introduction of various bans on noxious practices (e.g., cosmetic testing), or by instigating improvements in welfare standards of widely used practices they have publicised as unacceptable (e.g., phasing out of cages for layer hens in favour of barns or free-range) (for numerous other examples see [18,19]). Such beneficial reductions in animal suffering through vigorous Rights advocacy, which the advocates regard as too few, nevertheless may be seen to add weight to the minimising harm element of conscientiously deploying Strong Utilitarianism.

There are parallels here with Ecocentrism where it is not likely that full bans on human instigated disturbances to ecosystems, which include sentient beings, will be adopted. Nevertheless, it is important for environmental activists to pragmatically seek to have even a limited number of specified levels and standards adopted, as each step achieved paves the way for later successful negotiations. As with animal advocacy, there will also be benefits in educating the wider public.

In the likely event that animals will continue to be used for human purposes at some level and in some ways into the foreseeable future, it will be important for *Strong Utilitarian* principles to be applied conscientiously to harm-benefit assessments. The precise nature of the harms and the precise nature of the benefits, and their acceptability or otherwise at all levels of severity, not merely at the high extreme, must continue to be debated. Debate on these matters is important so that those involved in using animals for any purpose remain aware of their ethical responsibilities. Translating ethical principles into workable, pragmatic and easily understood animal welfare practices is equally important and is addressed in Section 8.

*Care Ethics or Ethics of Care* is a moral philosophy that prioritises relationships between individuals based on empathy and the responsibility to care for others in need, or to avoid harm. As such it challenges Utilitarian and Rights reasoning which tends to focus on abstract principles rather than personal connections, being intellectually driven, universalistic and collectively principled philosophies [20]. Care Ethics prescribes attentiveness to the needs of others and therefore is very well suited to supporting animals requiring help. It emphasises understanding the specific needs of individuals, building on relationship, acceptance of interdependence and a moral responsibility and obligation to care. By applying the core principles of Care Ethics, any tendency towards favouring known animals is managed in ways comparable to incorporating the ethical principle that all sentient beings deserve equal moral consideration, regardless of how close the relationship is with the care giver. The focus on a personal connection in providing care for animals, and avoiding harm, harmonises with the Animal Rights objective of banning practices regarded as exploitative.

## 3. The Emergence of Humane Societies and Laws Against Animal Abuse or Cruelty

The 19th Century saw the widespread emergence of charitable humane societies which can justifiably claim significant influence on curbing commonly observed animal abuse and cruelty. The details of only some of these developments are provided here.

Responding to previous failed attempts to pass anti-cruelty laws in the British Parliament, the first such humane organisation in the world was established in 1824; namely, the Society for the Prevention of Cruelty to Animals (SPCA) in England and Wales [21]. It received its Royal Warrant in 1840, becoming the RSPCA. Equivalent organisations were also established: the Ulster Society (1836), the Scottish Society (1839), and the Dublin Society (1840) [21]. Reflecting parallel interest in animals in the British Empire, SPCAs were established in New Zealand (1882) [22], and in a succession of Australian States, i.e., Victoria (1871), New South Wales (1873), South Australia (1875), Tasmania (1878), Queensland (1883), and Western Australia (1892) [23]. In 1981, the SPCAs in all states and territories amalgamated to form RSPCA Australia [23].

There were parallel developments in North America. Inspired by the emergence of the RSPCA in Britain, the American SPCA was established in New York in 1866. Also, the first law for the protection of animals was passed in 1866, and in the same year the organisation was granted the right to enforce anticruelty laws [24]. There are now SPCA’s widely distributed throughout the USA. In addition, there are ~51,000 other organisations committed to the humane treatment of animals [25]. In Canada, the first humane society was the Canadian SPCA, established in Montreal in 1869. Equivalent societies were soon established in Quebec (1870). Ottawa (1871), and Toronto (1873). Today, there are humane societies in major Canadian cities and in about 85 municipalities throughout the provinces [26].

In Europe, animal cruelty and abuse were actively discussed with a strong emphasis on legislation [27]. The first German animal protection society was established in Stuttgart in 1837, and by 1871 all German states except one had regulations against animal cruelty. Also at that time, Germany’s Animal Protection Society called for an expansion of laws so that the animal itself would be protected, and not because it was property, or because its mistreatment would cause public offence. The first Swiss law against animal cruelty was enacted in the Canton of Schaffhausen in 1842, and the first Swiss animal protection society was founded in Bern in 1844. France legally criminalised the public mistreatment of animals in 1850. In 1857, Sweden enacted its Criminal Law, which included statutes against animal cruelty, but unlike other European statutes at the time, the Swedish law penalised cruelty towards an animal regardless of its status as property. After the British Government passed the Cruelty to Animals Act 1876, the first in the world to regulate animal experimentation, vigorous debate ensued about regulating vivisection, particularly in Germany during the next decade. Dogs were the focus of an anticruelty law in Spain in 1877, and the humane society in Germany (1886) and societies as a group from Denmark, Norway and Sweden (1891), appealed to lawmakers to regulate slaughter practices to ensure that they would be humane.

From the time of their establishment, all these humane societies actively debated and sought the passing of legislation against cruel treatment of animals. Initially, they focussed on matters such as animal uses for draught or haulage purposes, in scientific experiments, slaughterhouse practices, in amusements such as fox hunting, bull baiting and cock fighting, and livestock management. Over time they also sought and were successful in broadening the offences and the species included. In so doing they collectively laid the foundations for 20th century developments in animal protection law, but still at that stage with a primary focus on cruelty.

## 4. Changes in Animal Welfare Science Thinking from 1980 to 2024

During the last 40 years scientific understanding of animal welfare has developed to the extent that Animal Welfare Science is now established as a distinct discipline. The original influential concepts have mostly been modified, extended or replaced. These changes are relevant to organisations that now seek to update their welfare policies, strategies and standards (for references see [4,28,29,30,31]).

This Section has two main purposes. The first is to show how the conceptual frameworks that were current at various times in the past influenced what were then regarded as valid ways of assessing and managing animal welfare. The second is to outline key features of contemporary understanding of animal welfare. Each subsection compares the prevalent ideas during the ‘Earlier period’ (1980s to late-1990s) to those in the ‘Later period’ (late-1990s to 2024).

### 4.1. Orientations of Animal Welfare Thinking

#### 4.1.1. Earlier Period: Competing Animal Welfare Orientations Vied for Primacy

*“Biological function”*: this was focused on growth, health, reproductive success and stress levels; physical, physiological, clinical and behavioural indices were preferred. Inferences about subjective mental states were strongly discouraged. The physical well-being of animals was the major focus.

*“Affective state”*: this focused on animals’ feelings, emotions and other subjective experiences; behavioural indices were mainly used to assess preferences, aversions and motivations; it was mostly concerned with potential subjective mental experiences of animals.

*“Natural living”*: this usually unfavourably compared welfare impacts of human-imposed restrictive conditions with better conditions in natural, wild environments; severe restrictions on animals’ ability to express natural behaviours raised most concern; numerous welfare challenges that arise in the wild were rarely mentioned.

These three orientations were often regarded by their proponents as competing schools of thought.

#### 4.1.2. Later Period: A Concept Integrating These Orientations Emerged

A more unified understanding became increasingly accepted. *“Biological function”* is now taken to include *“affective states”*, and *“affective states”* are recognised as products of *“biological function”*. Thus, the two are now seen to interact dynamically within the body operating as an integrated whole entity, and this understanding is regarded as fundamental to managing and improving the welfare of animals.

*“Natural living”* remains a useful benchmark against which to retrospectively or prospectively assess the welfare impacts of human-imposed restrictions on the behaviours animals can express, especially in intensively managed conditions. Included are impositions on farm livestock (e.g., chickens, sows), some companion pets (e.g., birds, cats, dogs), zoo animals (e.g., primates, big cats, elephants) and animals used for sport and recreation (e.g., dogs, horses). Encouraging behaviours that evolved in ancestral natural habitats is now strongly advocated.

### 4.2. Comparing Two Influential Systems for Assessing Animal Welfare

#### 4.2.1. Earlier Period: The Five Freedoms Framework

The first to address broader dimensions of animal welfare, this framework included subjective experiences, health status, behaviour and impacts of external circumstances. The specified purposes were for animals to be “free” from: the experiences of thirst, hunger, discomfort, pain, fear and distress; the states of malnutrition, disease and injury; and exposure to ambient extremes and behavioural restriction. Each “freedom” was aligned with a specific “provision”, which was a general statement of the practical measures needed to achieve the desired “freedom”.

The Freedoms framework was exceptionally influential due, at least partly, to the persuasive overall aim of being “free” of a wide range of welfare problems. But viewing the named affects as “bad” and expressing the remedy in terms of their elimination (being “free” of them) was ultimately recognised as biologically inaccurate and misleading. This is because thirst, hunger, pain and other such affects (e.g., breathlessness), have each been genetically embedded to motivate animals to engage in specific behaviours which are essential for survival. Also, the negativity and distinctive character of each affect are essential to create a sense of urgency to engage in very specific survival-critical behaviours. Thus, these affects can never be eliminated. However, they can be minimised to tolerably low levels that still elicit the required behaviours.

#### 4.2.2. Later Period: The Five Domains Model for Animal Welfare Assessment

The Model was devised in 1994 to comprehensively assess negative welfare impacts of RTT procedures on sentient animals. From 1997, the regulations governing approval for all RTT procedures conducted in New Zealand required Model welfare assessments to be conducted in the wide range of species utilised for these purposes. In addition to RTT animals, such Model assessments have now also been applied in other contexts, i.e., to farm, companion, working, sport, zoo, entertainment, “pest”, and wildlife animals, including possums, kangaroos and whales. The Model and its range of application have been regularly updated to include the continuing development of animal welfare science thinking.

The five domains are nutrition, physical environment, health, behavioural interactions and mental state. Factors considered in the first four domains give rise to affects which are assessed in the fifth (mental) domain. Thus, from the outset the Model integrated the “biological function” and “affective state” orientations. The negative affects generated by imbalances or disruptions within the body, captured mainly by the first three domains, are mainly those that elicit *internally generated*, *survival-related behaviours*. These affects include breathlessness, thirst, hunger, pain and others (see below). The fourth behavioural domain largely focuses attention on *externally generated*, *situation-related negative affects*. These reflect the animal’s perception of its external circumstances when interacting with its environment, other animals and humans; these affects include panic, frustration, loneliness, boredom and others (see below).

### 4.3. Adding Positive Welfare State Assessment to Negative Only Assessments

#### 4.3.1. Earlier Period: The Focus Was Primarily on Negative Welfare States

The motivating focus of animal welfare science at its inception was to identify, understand and minimise welfare problems, conceptualised in terms of negative physical, functional and/or experiential states. Research directed at solving these problems sought to minimise their negative impacts and led to numerous welfare improvements. Application of both the Five Freedoms framework and the Five Domains Model was motivated by these intentions.

#### 4.3.2. Later Period: Both Negative and Positive Welfare States Became the Focus

Acceptance that an animal’s welfare reflects what it experiences subjectively became widespread. Initially the focus was on specific negative affects which tend to be welfare-compromising (as noted above); then attention was given increasingly to specific positive affects which tend to be welfare enhancing. Extensive and rigorous neuroscience studies that clarified in each case the underlying affect-specific brain processes strongly supported this approach and added to its eventual widespread acceptance.

### 4.4. Adding More Affects for Consideration in Welfare Assessments

#### 4.4.1. Earlier Period: Additional Negative Affects Identified and Categorised

The negative affects listed for the 1994 Five Domains Model were thirst, hunger, anxiety, fear, pain and distress, distress being a generic term which included several other definable affects. During subsequent Model updating two categories were identified: (1) “internally generated, survival-critical negative affects” which include breathlessness, thirst, hunger, pain, nausea, dizziness, debility, weakness and sickness; and (2) “externally generated, situation-related negative” affects including anxiety, fear, panic, depression, frustration, anger, helplessness, loneliness and boredom.

#### 4.4.2. Later Period: A Wide Range of Positive Affects Identified

These affects include, but are not limited to: the quenching pleasures of drinking water; pleasures of food tastes, smells and textures; postprandial satiety; thermal, respiratory, olfactory, auditory and visual comforts; comfort and vitality of good health and fitness; in environmental and social interactions, feeling engaged, focussed, energised, socially bonded, parentally rewarded, excitedly playful and sexually gratified; feeling secure, protected, confident and in control.

The Model now includes reference to all these affects.

Further details of the Model and its applications are provided in Section 8.

### 4.5. The Importance of Keeping Animal Welfare Knowledge Up-to-Date

These observations illustrate the importance of remaining familiar with current scientifically supported understanding so that welfare policies and recommendations are truly up to date. Such updating is essential to give credibility to claims by organisations that their highest priority is the welfare of their animals in all aspect of their use and management. Regrettably, some of those currently at the top of sectoral organisations have not kept pace with these developments, and thus still rely on the more limited understanding available 20–30 years ago.

## 5. The Prelude to Scientists Supporting the Notion That Many Animals Are Sentient

*Animals Protection* legislation of the 1960s and 1970s had a strong focus on defining, preventing, detecting, and punishing cruelty. It characterised cruelty as willfully causing animals to suffer, specifying the causes as neglect, pain or injury; but it generally excluded accepted practices of farm animal management (including common painful husbandry procedures) and slaughter (e.g., [10,32]). In cruelty prosecutions, judges invariably relied on physical, clinical and other material evidence derived from ‘biological function’, because the prevalent view then was that the ‘affective experiences’ of animals could not be evaluated objectively [33]. However, that view is now changing (see Section 6).

As noted above, from the mid-1990s, the earlier findings on “affective state” were combined with those on “biological function”. This synthesis was greatly enhanced by innovative observations of the brain activities and regions that generate affective experiences (see Section 4.4). These were revealed by the then burgeoning and now well-established discipline of Affective Neuroscience [34]. This strengthened the new and fast developing conceptual framework that now coherently underpins the widely used Five Domains Model for guiding systematic and comprehensive assessment of animal welfare and its management (see [29] and Section 9).

These, and other parallel developments, led to emergence and consolidation of the now commonly held scientific view that animal welfare is primarily concerned with what animals’ experience subjectively. This acknowledgement by many Animal Welfare Scientists, and others, paved the way for the increasingly widespread declarations that all vertebrates and some other classes of animals are sentient.

## 6. Legal Recognition of Animal Sentience

Sentience in the context of animal welfare is the capability to consciously have subjectively negative and/or positive experiences or affects that matter to the animal [1,35]. They matter because negative experiences of significant intensity and/or duration can constitute suffering, and other experiences may be sufficiently positive for animals to find them rewarding [1]. The animal welfare scientists who focused on affective states, both before and after they were widely recognised to be closely integrated with biological function (Section 4), clearly understood that for affective states to be of welfare significance they must be experienced consciously in valance-specific ways, i.e., as negative or positive. However, initiatives that led to widespread legal recognition of welfare-aligned sentience did not occur until the cogent scientific support for it became much more detailed and widely accepted (see [1]).

Key international vehicles affirming animal welfare aligned sentience included the following: (1) the 27 countries of the European Union via the Treaty of Lisbon (2009), although the sentience-associated animal welfare implications of religious rites, cultural traditions and regional heritage were excluded [36]; (2) inclusion in a proposed United Nations Universal Declaration on Animal Welfare (2014), supported by 46 countries [37]; and (3) adoption by the ~180 member countries of the World Organisation for Animal Health (WOAH), via a statement recognising animal sentience in its Global Animal Welfare Strategy [38]. Following these initiatives, to date animal sentience has been asserted in the laws of a total of 36 countries, with partial support in eight others [39]. Of 50 countries that have not formally recognized animal sentience, ~40 have various forms of anticruelty laws [39].

Legally recognising sentience has broadened the range of welfare compromises courts can consider when hearing cases of cruelty [33]. These hearings frequently utilise expert opinions which evaluate the nature and seriousness of reported negative welfare impacts. For several decades the courts required statements about such impacts to be supported mainly by physical and/or clinical evidence, usually regarding commentary about animals’ subjective experiences as being scientifically unsupported anthropomorphic speculation. This orientation reflected the initially prevalent focus on biological function as the basis for understanding animal welfare (Section 4.1.1).

However, that has now changed due to the following progression of events: the scientifically validated integration of ‘biological function’ and ‘affective state’ thinking [1,40]; its inclusion in the Five Domains Model throughout the last 30 years [29]; the forensic use of the Five Domains Model for framing expert opinions [33]; and now, all of this reinforced by the legal recognition of animal sentience and the associated ability of animals to have negative and positive subjective experiences, or affects (see Section 4 and [33]). These developments have expanded the forensic scope of expert opinions in animal cruelty cases by formally allowing more detail about different types of suffering to be presented. On the basis of Canadian experience, it is recommended that courts should now recognise—as validly based—expert opinions that provide cogent evidence of untoward affective outcomes caused by ill-treatment, supplemented, as appropriate, by any relevant physical and/or clinical evidence if that is available [33].

## 7. Implementing Ethical Principles in National and International Law

It is not realistic to expect most members of the public to have a detailed understanding of, or interest in, the foundational moral principles, ethical guidelines and technical scientific details that determine, at a national level, what are or are not regarded as acceptable ways of treating animals [41,42,43,44,45,46].

Modern animal welfare law increasingly tends to refer to animals generically. As noted in Section 1, the introduction to this commentary, New Zealand’s Animal Welfare Act 1999 provides guidance on the meaning of “animal” by referring inclusively to specific classes and developmental stages, and, for the purposes of the Act, by excluding human beings [2].

**Figure 1:**
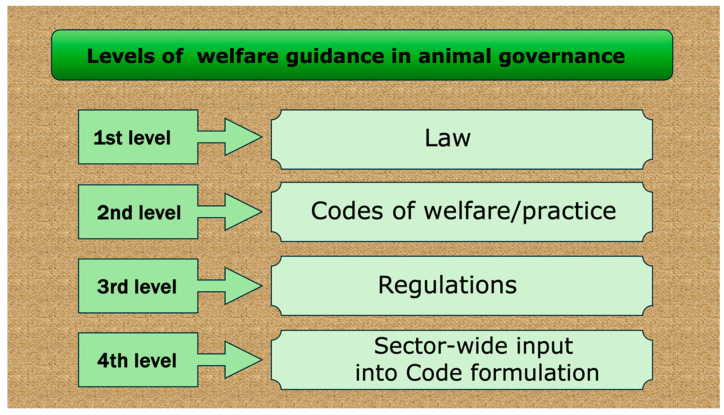
The level of governance: Level 1—Law; Level 2—Codes of Welfare/Practice; Level 3—Regulations; and Level 4—Sector-wide input into Code formulation.

Nationally, detailed welfare guidance to owners and persons in charge of animals is often provided at four levels which interact (Figure 1). For example, the New Zealand system [10].

*First level—Laws*: these outline each country’s welfare landscape in terms of defining animals, general principles, expectations of animal users, limitations, and, for serious breaches of the law, penalties that include imprisonment, heavy fines and/or banning from future animal contacts (e.g., [10,47,48,49,50]). It is common for the impact of each law to be strongly influenced by case law and the outcomes of appeals. Case law sets guidelines about how existing laws have been interpreted and applied, for example, in how habeas corpus laws (the right to freedom) are applied to sentient captive animals such as apes and elephants in suits brought by the Nonhuman Rights Project [51] and others.

*Second level—Codes of Welfare/Practice*: these describe in detail specific standards and how to achieve them (e.g., [52,53]).

*Third level—Regulations*: these identify less serious welfare infringements that nevertheless attract immediate financial penalties as fines (e.g., [54,55]).

*Fourth level—Sector-wide input into Code formulation*: here groups representing species-specific, process-specific and activity-specific animal sectors cooperate with expert advisory committees which have the formal role of ensuring that the final codes of welfare/practice comply with the law and any other relevant legal instruments (e.g., New Zealand [10,43,54,56]; Canada [53]; United Kingdom [57]. For example, there are two Ministerial Advisory Committees in New Zealand: the National Animal Welfare Advisory Committee, which deals with matters related to all animals in New Zealand, except those used in RTT, which is the responsibility of the National Animal Ethics Advisory Committee [10]. Overall, the membership of these committees includes animal welfare, biological, medical and/or veterinary scientists; also, sectoral experts; animal welfare advocates, laypersons, ethicists, educational experts, and others who may be seconded to assist with specific issues. A particular strength of both committees is that the Minister appoints each member in a personal capacity, not as an advocate for their affiliated organisation, so they may work more cooperatively as a team to provide comprehensively considered advice to the Minister.

Many countries’ animal welfare laws are additionally influenced by international federations and organisations, like the European Union or the WOHA. Recently, environmental and sustainability factors also influence the principles for law-making, as the balance in the overall ecosystem is being considered.

International animal welfare standards have been *developed* and *explained*. For example, a total of 16 welfare codes for terrestrial and aquatic animals have been prepared by the WOAH (previously the OIE) [58,59,60,61]. These codes have been approved by the WOAH’s ~180 current member countries. The European Food Safety Authority (EFSA) serving 27 European Union members has also *developed* and *explained* welfare standards for nine categories of farmed animals [62].

In addition, species-specific national standards prepared by others have been *adopted* for international use. For example, the International Federation of Horseracing Authorities (IFHA) with 60 members [63] largely based its “IFHA Minimum Horse Welfare Guidelines 2023” [64] on the New Zealand Thoroughbred Racing “Thoroughbred Welfare Assessment Guidelines” [65].

## 8. Translating Ethical Principles into Workable Animal Welfare Practices

Laws in areas of clear ethical interest are usually framed to highlight the values upon which they are based, motivated in the present context by humane concern for animal welfare. Such laws have several roles. Specifically in this case, to acknowledge that the proper care of animals is a “public good”. Also, to note that the legal provisions relate only to sentient animals, because they can suffer and can also have positive experiences. Usually included in such laws is general guidance on how contemporary welfare standards can be achieved and the ways they will be kept up to date. These outcomes are all accomplished via the four levels of governance mentioned above (Section 7).

Successful operation of such a law anticipates cooperation guided by Codes of Welfare/Practice, Humane Society advice, Ministry and industry information sheets and/or a personal commitment from animal owners in the country because, as with all laws, it is impossible to monitor compliance by everyone. Nevertheless, provision is made to detect non-compliance and to assess its cause. For example, causes may be unintentional or due to willful self-interest. Unintended non-compliance is often dealt with by education, or by recruiting help from friends, neighbours or others to assist with temporary crises. Flagrant infringements would be prosecuted.

**Figure 2:**
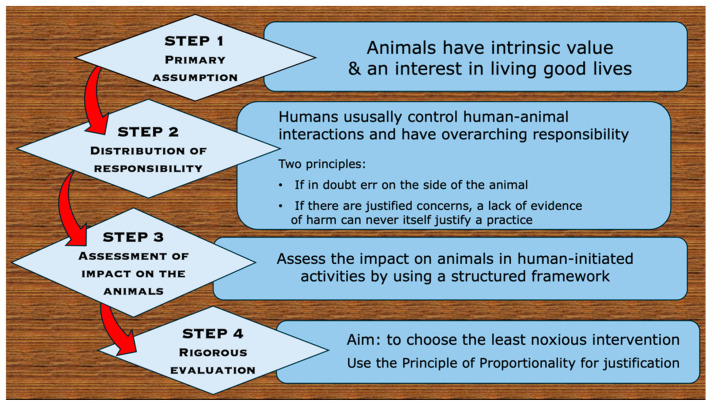
The four steps in applying ethical precepts.

Although the framing of such laws is based on ethical principles, the expression of those principles is more implicit than explicit. However, their expression can be made much more explicit when acting upon legal requirements to assess the acceptability of different practices in animal welfare terms. There are four interacting steps in this process (Figure 2).

*Step 1.* The process should always begin with a primary assumption that animals themselves have intrinsic value and have an interest in living good lives [46,66]. This circumvents unconscious assumptions of unlimited human primacy over animals, and it should generate a strong sense of respect for them. It also emphasises that whatever harm might be caused, it must comply with the Principle of Proportionality in being the minimum achievable. Yet, if possible, that should be even lower than the level of harm justified as proportionate to the pressing need [66].

*Step 2.* At this point it is important to acknowledge that, notwithstanding a respectful regard for the animals, such human-animal interactions are not equal. As humans are usually in control, they have an overarching responsibility for all the consequences of each interaction. Therefore, two implications of the Precautionary Principle must be applied: namely, (1) “If in doubt, err on the side of the animal”, and (2) “If there are justified concerns at empirical or other levels, a lack of compelling evidence of harm can never in itself justify a practice”.

*Step 3.* This involves utilising a structured and comprehensive framework for assessing the welfare impacts of animals used in human-initiated activities. The Five Domains Model for assessing and managing animal welfare is widely used for this purpose. It is based on the latest authenticated scientific understanding of animal welfare, is widely recognised and is described in Section 8.

*Step 4.* An equally rigorous evaluation must be conducted to assist in choosing the least noxious of the available interventions that might be used to achieve the desired purposes. This can be done by conscientiously outlining the current state of knowledge of the animals’ level of sentience, intelligence, basic needs, genetically unique sensory capabilities and behavioural requirements. Equally important is a factual account of the precise interventions it is proposed to implement and their anticipated welfare impacts. Once established, this information enables: (1) meeting the required justification of ‘proportionality’ (Section 9) that any harms are substantially lower in relation to the anticipated benefits, (2) consideration of the confidence attributable to that conclusion, and (3) a specific decision made about whether to proceed. Detailed record-keeping allows such decisions to be revisited if any of the weighted factors change.

These steps, recently outlined by Uldahl et al. (2022) [46], would clearly strengthen the practice of *Strong Utilitarianism* (Section 2) as described for guiding the humane use of animals [8,9]. It is important to note that these steps would be applied in all investigations that lead to the development or updating of welfare standards outlined in Codes of Welfare/Practice, Codes which provide the basis for animal welfare management in all sectors for which they have been prepared.

## 9. The Five Domains Model for Detailed Animal Welfare Assessment

The Five Domains Model is an integral part of Step 3 for giving effect to animal welfare laws, as described in Section 8. Several terms relevant to the Model have been helpfully defined [31]:

- *Assessment* is the process of judging or deciding on the nature and quality of an animal’s state of welfare based on available evidence.
- *Measurement* is the process of quantifying the characteristics of an animal’s welfare so those characteristics can be compared with previously established standards;
- *Auditing* is an on-site verification activity, such as an inspection or examination, to ensure compliance with established welfare standards and requirements; and
- *Monitoring* involves observing and checking the progress or quality of welfare over a period, maintaining regular surveillance, and systematically reviewing and reporting on the findings.

The primary purposes of the Model are to *assess* identifiable features of animal welfare, and by *monitoring* them, to direct practical management activities towards areas that need improvement and/or to identify those areas that are already regarded as acceptable or better.

As an emphasis on affects has been integral to the animal welfare reasoning deployed throughout the 30-year evolution of the Model [29], “assessment” of welfare has always been the preferred terminology, because the notion of “measurement” is both logically and practically flawed given the focus on affective experiences [40].

**Figure 3:**
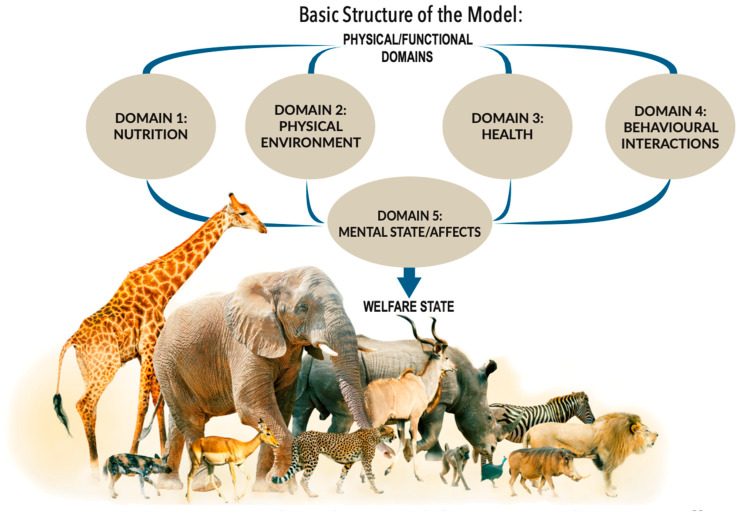
The basic structure of the 2020 Five Domains Model (reproduced with permission [29]).

The Model is structured to guide systematic and comprehensive assessments of the welfare of *individual* animals, whether they are alone or in groups (Figure 3). Comprehensiveness in this context refers specifically to the thoroughness of assessments of the focal individuals. It does not include, as Hampton et al. (2023) implied it should [67], unintentional, indirect or collateral negative welfare impacts on other animals. If any animals impacted indirectly are of interest (e.g., [68]), they can and should be evaluated separately using the Model.

The first four Domains, collectively designated *physical/functional*, are 1 Nutrition, 2 Physical Environment, 3 Health, and 4 Behavioural Interactions with (a) the environment, (b) other animals, and (c) human beings [29]. Listed against each of these Domains are specific examples of factors that may lead to compromised welfare or to acceptable-to-good welfare (readers are recommended to access the detailed Five Domains Model Infographic (2020) at https://app.box.com/s/88rakysmtmseh4eha21fx4jdy6ps47fp, accessed on 9 March 2025). Assessments that refer to the listed factors within each of these four Domains draw attention to possible remedies such as specific corrective activities, including the provision of different foods, shade and/or shelter, veterinary attention, sufficient space and environmental variety, the presence of congenial other animals, and well-informed care and attention from people with whom they interact.

The indices for each of these factors are mostly well-understood physical, anatomical, biochemical, physiological, clinical, behavioural and other signs. Species differences occur due to their unique genetics (e.g., a dog is not a cat or a cow or a bird), somewhat modified by impacts of specific differences in the ancestral bioecological niches in which they evolved, or by purposeful contemporary breeding strategies. Those not familiar with species-specific signs should seek advice from well-informed others. These indices provide the objective information which, when aligned with Domain 5, Mental State, enables cautious inferences to be made regarding the animals’ likely experience of specific affects associated with each factor ([29] illustrated by the Five Domains Model Infographic, 2020).

These affects are not measured. Their presence is cautiously inferred based on established affective neuroscience and indicative behaviours. The affects have several roles in the Model: (1) their wide range highlights the breadth of welfare factors that need to be considered for the assessments of individual animals to be comprehensive; (2) each factor plus its aligned specific affect within each Domain, needs to be identified separately, and then they all need to be considered for whatever specific corrective actions may be required for each one; and (3) the existence of both negative and positive affects supports the dual objectives that welfare management should aim both to minimize negative affects to at least tolerably low levels and to provide opportunities for animals to have positive experiences.

The success or otherwise of corrective actions in each animal should be *monitored* by repeated assessments of the observable signs related to individual affects, systematically undertaken within each of the first four Domains in sequence. Thus, affective impacts considered in Domain 5 are those aligned with factors identified via the first four Domains. Note that each factor/affect is evaluated separately.

Aggregation of affects must be avoided because there is no cogent basis for assessing the relative impacts of, or interactions between, different affects, whether negative or positive [1,29,66,69]. This rules out numerical manipulations to give overall scores. Indeed, these uncertainties undermine the validity of any aggregated scores, no matter how many factors and related affects are included [69], and no matter how repeatable the scoring is by individuals or groups of assessors might be [66]. Thus, all notions of a Quality-of-Life Index or an Overall Animal Welfare Score are deeply flawed and unreliable [1,69], no matter how intuitively engaging they are, or their hoped-for usefulness might be [70]. This also rules out notions of measurably compensating animals for periods of overall poor welfare by period of overall good welfare. During the 30 years since the first version of the Model was published [71], it’s breadth and content have markedly increased as knowledge improved (Five Domains Model Infographic 2020), thereby providing an alternative approach to aggregation.

Mandatory record keeping is commonly grounded in law as part of regulatory governance, and to provide an incentive for and a check on compliance levels. It can also have educational, research and developmental purposes. All these features need to be based on up-to-date conceptual frameworks, be informative and easy-to-use.

A Welfare Action Worksheet could be used. It would identify any specific welfare compromising factors that require attention in each of the first four Domains, for example, designating them as ‘urgent’ (needing immediate attention), ‘serious’ (needing attention in <3 days), ‘pressing’ (needing attention in <3 weeks) or ‘of concern’ (needing attention in <3 months). Against each would be a note of the intended remedy. Regarding welfare enhancing factors that generate positive experiences, for each of Domains 1 to 4 those that are both available and utilized would be listed, and others that could be introduced would be described and the timing of their introduction noted.

A recently described, independently developed, smartphone-based welfare assessment App, the Mellorater [31], complies with the foundational guidelines of the Five Domains Model (as just described) and employs a simplified checklist of questions to cover a broad range of factors that are known to influence animal welfare outcomes.

## 10. The Principle of Proportionality

A purposeful and detailed evaluation of the extent and nature of welfare compromise to be imposed on the animals by planned human uses is described in Section 7 Step 3.

The Principle of Proportionality, a tenet widely embedded within the German legal system, is aligned with Utilitarianism, because the focus of proportionality is on the avoidance of disproportionate harms. This focus helped to shape the Animal Protection Act 1972 in West Germany, and current European Union animal welfare legislation also follows the Principle of Proportionality. A detailed account of the ethical, legal and operational contexts of the Principle of Proportionality as applied to RTT uses of animals is available [66].

At least two key questions should be addressed when assessing the compromise likely to be experienced by the animals:

1. What compromises can never be justified?
2. Are there alternatives that cause less compromise?

An obvious answer to the first question would be that any procedures or actions that would cause unremittingly intense and protracted pain, breathlessness, nausea, dizziness, panic, terror, or other such noxious experiences, which would undoubtedly constitute extreme suffering, would never be justified. However, this answer might be challenged by the view that humans can inflict any levels suffering on animals if the human benefit is construed to be great enough. Such an extreme application of Utilitarianism, notably lacking in empathy, does not allow for the beneficial constraining influences of co-existing ‘Reverence for Life’, ‘Animal Rights’ and ‘Animal Care’ ethical perspectives. To achieve greater insight during ethical evaluations it is usual to deploy more than one ethical theory.

Such constraining influences may contribute to the questioning of a perceived over emphasis on human advantage when deploying Utilitarian reasoning to justify the high “ethical cost” of some biomedical research [72]. Likewise, over the past several decades in the agricultural sector, there have been well-known evidence-based challenges to, for example: close confinement systems for battery caged layer hens, densely packed barn-reared broiler chickens, and the extreme movement restrictions in sow stalls or crates; long-distance transport; stressful handling of livestock in meat works; unreliable efficacy of preslaughter stunning and/or exsanguination; conducting husbandry procedures involving cutting, constriction, crushing, compression, cautery and cryo-cautery in numerous species without pain relief; noxious effects of trapping and poisoning of so called “pest” animals; and more. Indeed, virtually all areas of animal agriculture have been affected by advances in understanding of animal welfare and its management, communicated to each sector in revised Codes of Welfare/Practice, and by other means mentioned above. However, not all countries have adopted the improvements. This perceived reluctance provides a further reason for strengthening the understanding of the mechanisms described here by which Utilitarian reasoning can be put into effect to benefit the animals. Other sectors where negative welfare impacts are currently being critically evaluated include animals used in sport and other entertainments (e.g., [73,74,75]).

The second question applies to all levels of compromise from the lowest to the highest. At the very highest level, it is when a search for a less compromising alternative fails that the proposed extreme imposition would be declared unacceptable. Even at very low levels of compromise, alternatives that have even lower negative impacts should be sought. Moreover, seeking alternatives at any level, may reveal options that could be beneficially applied at other levels. It is an ethical imperative to ask this question, whatever the level.

## 11. Concluding Comments

Clearly, concerns about animal welfare have ethical foundations. Our undoubted domination of the sentient animals with which we interact has therefore raised ethical questions. These are related to whether this domination is provisionally acceptable, and if so, how it’s various purposes may be justified, and given animals’ capacity to suffer, what safeguards should be applied on their behalf. Utilitarianism, i.e., conscientiously maximising the ‘benefits’ and equally conscientiously minimising the ‘harms’, has been widely adopted internationally to underpin animal welfare policies and practices at a national level, and in federal systems, at state or provincial levels. However, the ethical landscape surrounding animal welfare includes Animal Rights constructs framed as obligations which, at their extreme, totally oppose any use of animals for human purposes. Likewise, Animal Care constructs based on empathy, harmonise with these Rights objectives. These views should be acknowledged as legitimate concerns, and they should be valued as constraining callous treatment of animals which may have been justified by an overemphasis on human self-interest under the deceptive guise of weak Utilitarian reasoning.

Offered here are examples of how ethical reasoning, and its operational consequences, can be made visible at every stage of developing, introducing and operating infrastructures for managing animal welfare nationally. Imperative to this endeavour is an up-to-date science-based understanding of animal welfare and how it may be assessed and managed. Influential individuals whose understanding is 20–30 years out of date delay necessary welfare improvements. Once these infrastructural features are established, based on up-to-date knowledge, there is clear evidence of their dissemination and recommended adoption internationally.

## Data Availability

Not applicable.
